# *Amblyomma imitator* Ticks as Vectors of *Rickettsia rickettsii*, Mexico

**DOI:** 10.3201/eid1608.100231

**Published:** 2010-08

**Authors:** Karla A. Oliveira, Adriano Pinter, Aaron Medina-Sanchez, Venkata D. Boppana, Stephen K. Wikel, Tais B. Saito, Thomas Shelite, Lucas Blanton, Vsevolod Popov, Pete D. Teel, David H. Walker, Marcio A.M. Galvao, Claudio Mafra, Donald H. Bouyer

**Affiliations:** University of Texas Medical Branch at Galveston, Galveston, Texas, USA; 1 Current affiliation: Federal University of Vicosa, Vicosa, Brazil.; 2 Current affiliation: Superintendency of Control of Endemic Diseases, São Paulo, Brazil.; 3 Current affiliation: Hospital and Clinic Oca, Monterrey, Nuevo Leon, Mexico.; 4 Current affiliation: Texas A&M University, College Station, Texas, USA.; 5 Current affiliation: Federal University of Ouro Preto, Ouro Preto, Brazil.

**Keywords:** Ticks, Rickettsia rickettsii, rickettsia, Amblyomma imitator, Rocky Mountain spotted fever, ultrastructure, vector-borne infections, Mexico, dispatch

## Abstract

Real-time PCR of *Amblyomma imitator* tick egg masses obtained in Nuevo Leon State, Mexico, identified a *Rickettsia* species. Sequence analyses of 17-kD common antigen and outer membrane protein A and B gene fragments showed to it to be *R*. *rickettsii*, which suggested a potential new vector for this bacterium.

*Rickettsia rickettsii* is a gram-negative, obligate, intracellular bacterium and the cause of Rocky Mountain spotted fever (RMSF). In Mexico, its transmission has been attributed to *Rhipicephalus sanguineus* and *Amblyomma cajennense* ticks.

*Amblyomma imitator* has close affinity with *A*. *cajennense* and was formerly confused with this species. These ticks’ distributional range extends from southern Texas, southward through Mexico (where they are widely sympatric with *A. cajennense* ticks) into Central America (*1*). In this study, we isolated and characterized *R. rickettsii* from *A. imitator* from Mexico by using molecular methods.

## The Study

Males, females, and nymphs of 5 tick species (*A*. *imitator*, *Rhipicephalus microplus*, *Dermacentor variabilis*, *Rh*. *sanguineu*s, and *Ixodes* species) were collected in Nuevo Leon State, Mexico (24°50′N, 100°4′W) in 2007 (females, males, and nymphs) by using dry ice traps (*1*). Ticks were identified by using morphologic keys as defined by Kohls (*2*) and Keirans and Durden (*3*) and maintained in the laboratory at the University of Texas Medical Branch (Galveston, TX, USA) according to methods described by Brossard and Wikel (*4*). The colonies were maintained at 22°C, under a 14-hour light/10-hour dark photoperiod. Ticks were held in 16-mL glass vials (Wheaton Glass, Millville, NJ, USA) with a mesh top over a supersaturated solution of potassium nitrate. Larvae and nymphs obtained blood meals from mice, and adults were fed on pathogen-free rabbits. Only the *A. imitator* colony was successfully established.

Real-time PCR analysis of eggs laid by 1 full generation of laboratory-reared females identified rickettsial DNA in 2 egg masses. DNA from 5 egg masses laid by different females was extracted by using a DNeasy Kit (QIAGEN, Valencia, CA, USA). Real-time PCR was performed by using *Rickettsia* spp.–specific primers CS5A and CS6 (*5*) for amplification of a 150-bp fragment of the citrate synthase gene in an iCycler thermocycler (Bio-Rad, Hercules, CA, USA) as described (*5*). *R*. *australis* DNA and water served as the positive and negative controls, respectively, and serial dilutions of a plasmid that contained the *R*. *prowazekii* citrate synthase gene were used as standards.

We selected egg masses for isolation of the rickettsial agent in Vero cells by using shell vials as described (*6*). The shell vials were incubated at 34°C and monitored daily by Diff-Quik staining (Dade International Inc., Miami, FL, USA) for rickettsiae. Slides that contained >4 rickettsiae were considered positive. The monolayer from the corresponding shell vial was removed manually, placed in a T-25 flask containing Vero cells (in Dulbecco minimal essential medium containing 3% heat-inactivated bovine calf serum). Samples were then placed in 150-cm^2^ flasks containing Vero cells for propagation of the agent.

For characterization of isolates, partial sequences of *Rickettsia*-specific genes were amplified and analyzed. Nested PCR was performed by using primers 17K3 and 17K5 (*5*) for the first reaction and 17KD1 and 17kD2 (*7*) for the second reaction of amplification of 17-kD common antigen (*htrA*) gene fragments. For amplification of an outer membrane protein B (*ompB*) gene fragment, primers 120-M59 and 120–807 were used (*8*). Seminested PCR with primers 120-M59 and 120–807 for the first reaction and 120–607 and 120–807 for the second reaction was performed for 1 of the samples (*8*). An *ompA* fragment was amplified by using primers Rr190.70F and Rr190.602R (*9*). For 1 of the samples, a seminested PCR was necessary; primers Rr190.70F and Rr190.701R (*10*) were used for the first reaction and Rr190.70F and Rr190.602R for the second reaction.

Fragments were cloned into pCR4-TOPO (Invitrogen, Carlsbad, CA, USA), and plasmids from selected clones were sequenced >3× by using universal primers M13F and M13R. Nucleotide sequences were edited with SeqMan (www.dnastar.com/t-sub-products-lasergene-seqmanpro.aspx) and used for BLAST analysis (*11*). Amplification of the *ompB* fragment was achieved for only 1 of the samples. This finding was probably the result of a mutation at the site of primer annealing because amplification of another region of the gene was possible.

Analysis of sequences obtained from the *htrA* gene fragments (434 bp) amplified from both samples showed 99% identity with spotted fever group *Rickettsia* sequences, including the *R*. *rickettsii* sequence from a fatal case of RMSF in southwestern Mexico, Yucatan State (GenBank accession no. DQ176856.1) (*12*), with only a 1-nt difference. Analysis of the partial sequence of *ompB* (856 bp) and *ompA* (533 bp) genes showed 100% identity with *R*. *rickettsii* Sheila Smith strain (CP000848.1). Sequences obtained in this study were submitted to GenBank under accession nos. GU723476 and GU723477 for *htrA* fragments, GU723478 and GU723479 for *ompA* fragments, and GU723475 for the *ompB* fragment.

For detection of rickettsiae in *A*. *imitator* ticks, salivary glands, midguts, and ovaries were dissected from unfed adult ticks and fixed in modified Ito fixative (*13*), postfixed in 1% osmium tetroxide for 1 h, stained en bloc with 2% aqueous uranyl acetate for 20 min at 60°C, dehydrated in ethanol, and embedded in epoxy resin (Poly/Bed 812). Ultrathin sections were cut with an Ultracut S Ultramicrotome (Reichert, Vienna, Austria), placed on copper grids, and stained with lead citrate. Ticks were tested for *Rickettsia* spp. by nested PCR with primers 17K3/17K5 and 17KD1/17KD2, and sections of organs from PCR-positive samples were examined in a Philips CM-100 electron microscope (FEI, Hillsboro, OR, USA) at 60 kV.

Single rickettsial cells were found in highly vacuolated cytoplasm of midgut epithelial cells of 1 male tick (no. 5) (Figure, panel A). Cells had typical ultrastructure for gram-negative bacteria and were surrounded by 2 trilaminar membranes (Figure, panel B): an inner cytoplasmic membrane and an outer cell wall membrane. Their size was 0.6 × 0.2 μm. Rickettsial organisms were not visualized in either salivary gland or ovaries probably because of a low number of rickettsiae, as suggested by the fact that detection of rickettsial DNA by PCR required a nested PCR.

**Figure:**
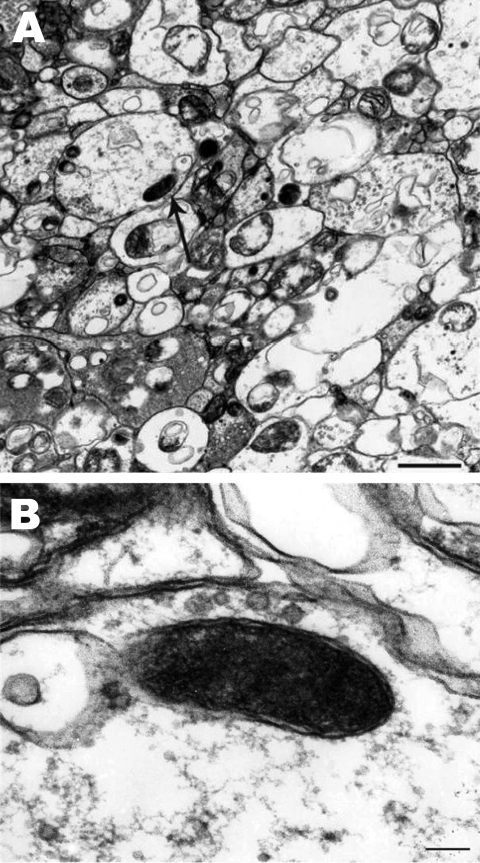
*Rickettsia rickettsii* (arrow) in a midgut cell of an *Amblyomma imitator* tick (A). The trilaminar cell wall is separated from the cell membrane by the periplasmic space (B). Scale bars = 0.1 µm.

## Conclusions

This study demonstrated that *R*. *rickettsii* was present in egg masses of *A*. *imitator* ticks. Because eggs were laid by field-collected, unfed, adult ticks that were fed in the laboratory on pathogen-free rabbits for 1 full generation, *R*. *rickettsii* in eggs documents their transovarial transmission by naturally infected ticks and suggests a role for this tick species in the maintenance of *R*. *rickettsii* in nature.

Because hosts of *A*. *imitator* ticks are various species of birds and mammals (*3*), the notable finding of this study is the potential participation of *A*. *imitator* ticks in a zoonotic cycle of *R*. *rickettsii*. According to Kohls (*2*), RMSF transmission studies performed with supposed *A*. *cajennense* ticks by Parker et al. (*14*) were actually performed with *A*. *imitator* ticks, which suggests that this species of tick could be a vector of *R*. *rickettsii*. On the basis of the results of our study and the aggressive nature of the tick for humans, we suggest that *A*. *imitator* ticks are a potential vector or at least involved in maintenance of *R*. *rickettsii* in nature in Mexico.
